# Systematic reviews of observational studies of risk of thrombosis and bleeding in urological surgery (ROTBUS): introduction and methodology

**DOI:** 10.1186/2046-4053-3-150

**Published:** 2014-12-23

**Authors:** Kari AO Tikkinen, Arnav Agarwal, Samantha Craigie, Rufus Cartwright, Michael K Gould, Jari Haukka, Richard Naspro, Giacomo Novara, Per Morten Sandset, Reed A Siemieniuk, Philippe D Violette, Gordon H Guyatt

**Affiliations:** Department of Urology, Helsinki University Central Hospital and University of Helsinki, Helsinki, Finland; Department of Public Health, University of Helsinki, Helsinki, Finland; Department of Clinical Epidemiology and Biostatistics, McMaster University, Hamilton, ON Canada; School of Medicine, University of Toronto, Toronto, ON Canada; Department of Epidemiology and Biostatistics, Imperial College London, London, UK; Department of Urogynaecology, St. Mary’s Hospital, London, UK; Department of Research and Evaluation, Kaiser Permanente Southern California, Pasadena, CA USA; Department of Urology, AO Papa Giovanni XXIII, Bergamo, Italy; Department of Surgical, Oncological, and Gastroenterological Sciences, Urology Clinic, University of Padua, Padua, Italy; Department of Haematology, Oslo University Hospital, Oslo, Norway; Institute of Clinical Medicine, University of Oslo, Oslo, Norway; Department of Medicine, University of Toronto, Toronto, ON Canada; Department of Surgery, Division of Urology, the University of Western Ontario, London, ON Canada; Department of Medicine, McMaster University, Hamilton, ON Canada; Michael G. DeGroote Institute for Pain Research and Care, McMaster University, Hamilton, ON Canada

**Keywords:** Baseline risk, Bleeding, Hemorrhage, Modeling, Risk of bias, Surgical complications, Thromboprophylaxis, Thrombosis, Urology

## Abstract

**Background:**

Pharmacological thromboprophylaxis in the peri-operative period involves a trade-off between reduction in venous thromboembolism (VTE) and an increase in bleeding. Baseline risks, in the absence of prophylaxis, for VTE and bleeding are known to vary widely between urological procedures, but their magnitude is highly uncertain. Systematic reviews and meta-analyses addressing baseline risks are uncommon, needed, and require methodological innovation. In this article, we describe the rationale and methods for a series of systematic reviews of the risks of symptomatic VTE and bleeding requiring reoperation in urological surgery.

**Methods/design:**

We searched MEDLINE from January 1, 2000 until April 10, 2014 for observational studies reporting on symptomatic VTE or bleeding after urological procedures. Additional studies known to experts and studies cited in relevant review articles were added. Teams of two reviewers, independently assessed articles for eligibility, evaluated risk of bias, and abstracted data. We derived best estimates of risk from the median estimates among studies rated at the lowest risk of bias. The primary endpoints were 30-day post-operative risk estimates of symptomatic VTE and bleeding requiring reoperation, stratified by procedure and patient risk factors.

**Discussion:**

This series of systematic reviews will inform clinicians and patients regarding the trade-off between VTE prevention and bleeding. Our work advances standards in systematic reviews of surgical complications, including assessment of risk of bias, criteria for arriving at best estimates of risk (including modeling of timing of events and dealing with suboptimal data reporting), dealing with subgroups at higher and lower risk of bias, and use of the Grading of Recommendations Assessment, Development and Evaluation (GRADE) approach to rate certainty in estimates of risk. The results will be incorporated in the upcoming European Association Urology Guideline on Thromboprophylaxis.

**Systematic review registration:**

PROSPERO CRD42014010342.

**Electronic supplementary material:**

The online version of this article (doi:10.1186/2046-4053-3-150) contains supplementary material, which is available to authorized users.

## Background

### Introduction

Venous thromboembolism (VTE), which includes deep vein thrombosis (DVT) and pulmonary embolism (PE), represents a serious, and sometimes fatal, complication of urological surgery. A systematic review and meta-analysis including gastrointestinal, urological, gynecological, and general surgical procedures has demonstrated that pharmacological prophylaxis decreases the risk of VTE in surgical patients by approximately 50% but increases the risk of post-operative major bleeding by approximately 50% [1]. The decision to use pharmacological prophylaxis therefore presents a trade-off between a reduction in VTE and an increase in bleeding.

The crucial issue in making this decision is the risk of VTE and bleeding in those not receiving anticoagulants, which we will refer to as ‘baseline risk’. Baseline risks, in the absence of prophylaxis, for VTE and bleeding are known to vary widely between urological procedures [2], but their magnitude is highly uncertain. In patients with a high risk of VTE and a low risk of bleeding, a 50% reduction in VTE represents substantial benefit (for instance, from a baseline risk of 10% to 5%). In a patient with a low baseline risk of bleeding (for instance, from 0.2% to 0.3%), the balance of benefits and harms clearly favors prophylaxis. The situation is reversed in patients whose risk of VTE without anticoagulation is low and whose bleeding risk is high. A small reduction in VTE will not warrant the substantial increase in bleeding. In patients whose risk of VTE and bleeding are similar, the decision will depend on the relative value patients place on avoiding VTE and avoiding bleeding.

Thus, the decision regarding thromboprophylaxis is critically dependent on the baseline risks of both VTE and bleeding. Reliable estimates of these risks require systematic summaries of the best available evidence, which have thus far been unavailable [3]. Consequently, the approach to thromboprophylaxis related to urological surgery is an area with marked practice variation, both within and between countries [4, 5].

Our series of systematic reviews aims to fill this critical gap in knowledge by addressing the risks of VTE and bleeding requiring reoperation in patients undergoing urological surgery but not receiving prophylaxis. Each review summarizes the evidence regarding the frequency of VTE and bleeding requiring reoperation for procedures for diseases of the urinary tract and (male) genital system. In this article, we outline the specific methods used in our reviews and familiarize readers with the methodology of systematic reviews and meta-analysis of observational studies of complications and their strengths and limitations.

### What are systematic reviews of baseline risk, and why are they important but challenging to perform?

Systematic reviews are summaries of a body of evidence, and meta-analysis is a quantitative, statistical method to summarize the results. Preparing a systematic review requires a number of decisions including determining the focus; identifying, selecting, and critically appraising the relevant primary studies; collecting and synthesizing the relevant information; and drawing conclusions from the evidence. Typically, investigators addressing questions of therapy frame their research questions in accord with the Patient, Intervention, Comparator and Outcome (PICO) format with associated eligibility criteria [6]. This facilitates the formulation of an answerable question from which estimates of effect can be derived.

Conventional systematic reviews that compare one treatment against another or against a non-treatment control are common and the methods are well established [6]. Systematic reviews and meta-analyses addressing baseline risks (that is, risk in the absence of intervention, in this case antithrombotic prophylaxis—of important adverse outcomes, in this case VTE or bleeding requiring reoperation) are sorely needed and require methodological innovation [7]. The rates of events in the absence of prophylaxis, required for estimation of benefits and risks, can come only from systematic reviews of the relevant studies. Nevertheless, few such systematic reviews have been undertaken in any area of surgery [8] and none in urology [3].

One reason for the dearth of such reviews is the multiple challenges they present. First, indexing of observational research is less well established than for randomized controlled trials (RCTs), making it harder to identify the relevant studies [9]. Second, surgical studies often focus on disease-related outcomes (for instance, radical prostatectomy papers usually report prostate cancer-specific outcomes) but typically not on generic complications (including VTE and bleeding requiring reoperation).

Third, even when articles report risks of VTE and bleeding, key information is often missing. Such information includes the use of prophylaxis (usually varying among patients), the timing of the events of interest, and diagnostic criteria for the events of interest. Fourth, risk of bias criteria, as well as criteria for overall certainty in estimates [10], well established for reviews of therapeutic trials are controversial in studies of baseline risk [7]. Fifth, the choice of studies to use for best estimates (e.g., synthesis from all studies or only from those at lowest risk of bias) is uncertain [10]. Sixth, the incidence of VTE has changed over time due to advances in surgical technique and care (e.g., early mobilization) [11–13].

In these reviews, we address these challenges with the goal of providing precedents for optimal methodology. For the overall certainty in estimates of effect, we used the Grading of Recommendations Assessment, Development and Evaluation (GRADE) system, which has provided detailed methodological guidance for systematic reviews [14], and initial standards for the assessment of baseline risk [7].

## Methods/design

### Eligibility criteria

We included studies published in English language medical journals that enrolled adult male or female patients undergoing procedures for diseases of the urinary tract and (male) genital system, including kidneys, ureters, bladder, prostate, seminal vesicles, urethra, scrotum, testicles, penis, and vagina (Additional files 1 and 2). Reasoning that very small studies are likely to be published only if they show anomalous results, we included only studies with at least 50 adult patients per urological procedure to decrease risk of bias.

Although RCTs provide estimates of effect of treatment with the lowest risk of bias, populations enrolled are usually highly selected and RCTs are therefore, with respect to estimating baseline risk, limited in terms of generalizability [15, 16]. Hence, observational studies of unselected patients undergoing urological surgery are likely to be the best source of estimates of VTE and bleeding risk. Because baseline risk has changed over time [11–13], we included studies that recruited all or a majority of participants after the year 2000. Because the complication estimates depend on the length of follow-up, we included only studies that clearly defined the time period of follow-up (up to 3 months).

Finally, we included only studies that reported at least one absolute estimate of risk of the patient important outcomes of interest (fatal PE, symptomatic PE, symptomatic DVT, symptomatic VTE, fatal bleeding, and bleeding requiring reoperation). Absolute estimates include the percentage (e.g., 1.0%), proportions (e.g., 0.02), natural units or natural frequency (e.g., 3 in 1,000 patients), and natural frequency per time (e.g., 3 in 1,000 patient years) but do not include relative estimates, such as risk ratio or odds ratio.

### Literature search, study selection, application of eligibility criteria, and data abstraction

We used two search strategies. First, we used the MEDLINE database to search for potentially eligible articles published from January 1, 2000 until April 10, 2014. A combination of keyword and medical subject headings search included the ‘urological procedures’ (more than 90 different urological procedures) term family combined with the ‘thrombosis’ term family (Additional file 1) as well as the ‘urological procedures’ term family combined with the ‘bleeding’ term family and the prognosis sensitivity filter (Additional file 2). We included articles not in the search but known to the experts in the panel. Finally, we identified further original articles by reviewing the reference lists of included systematic reviews (none of the systematic reviews summarized any of the outcomes of interest of our series).

Two reviewers evaluated titles and abstracts from the search and identified potentially eligible studies. We obtained the full articles of these potentially eligible titles and abstracts, and two reviewers assessed the full texts to make final judgments regarding eligibility. Similarly, two reviewers independently abstracted data including outcomes, study characteristics, and risk of bias. A clinician-methodologist adjudicator resolved disagreements regarding eligibility or study characteristics. Finally, we sent our consensus data extraction to the original authors of each article for confirmation or correction. When needed, we also asked authors to clarify details regarding thromboprophylaxis, surgical technique (such as pelvic lymph node dissection), as well as other missing or unclear information.

### Outcomes

Primary outcomes were the absolute risks of symptomatic VTE and bleeding requiring reoperation (including exploration and angioembolization). Secondary outcomes were the absolute risks of fatal PE and fatal bleeding. All outcomes were extracted and analyzed separately for each procedure (see ‘Analysis’ section).

We also extracted the length of follow-up regarding VTE and bleeding outcomes. When authors presented the frequency of events at more than one time point, we recorded the number of events up to 3 months and used the absolute risk closest to 4 weeks (our primary outcome). We did not collect data on events of questionable importance to patients [17], in particular asymptomatic DVT, changes in hemoglobin levels, or amount of estimated blood loss during the operation. Transfusion rates were not recorded primarily due to expected large differences in clinical practice that would make it difficult to estimate the trade-off of thrombosis and bleeding.

### Final selection of eligible studies (risk of bias and outliers)

We assessed design features that could potentially bias the estimates of VTE or bleeding risk. These include the representativeness of the recruited patient population, study type, losses to follow-up, explicitness of criteria for VTE diagnosis, thromboprophylaxis documentation, and data source (Table 1). We also collected information on several other characteristics of the articles and their study populations that may be predictive of VTE or bleeding requiring reoperation (Table 2).

**Table 1:** **Design features considered for assessment of risk of bias**

Domain	Lower risk of bias	Higher risk of bias
Sampling and representativeness of the population	Consecutive patient recruitment or administrative database with random sampling	Non-consecutive patient recruitment or administrative database with non-random sampling
Study type	International multicenter; multicenter in one country; single center, not single surgeon	Single surgeon series
Source of information	Data abstracted by investigators from patient charts	Administrative database information
Thromboprophylaxis documentation	Reporting of patients’ thromboprophylaxis	No reporting of patients’ thromboprophylaxis
Diagnostic criteria	Objective confirmation of symptomatic venous thromboembolism	No objective confirmation of symptomatic venous thromboembolism
Loss to follow-up	Less than 20% loss to follow-up	20% or more loss to follow-up

**Table 2:** **Characteristics assessed**

Characteristics	
Year of publication	Patient recruitment (first and last year)
Source of sampling^a^	Study type^d^
Country/countries	Multinational (yes/no)
Urological procedure(s)	Total number of patients
Gender distribution	Age (mean/median/threshold)
Proportion of patients with malignant disease	Use and extension of pelvic lymph node dissection
Patient use of mechanical thromboprophylaxis^b^	Patient use of anticoagulants^e^
Patient use of aspirin or other antiplatelet drugs^c^	Patient use of both mechanical and aspirin/anticoagulants^b,c,e^

^a^Either retrospective case series, register/administrative database, or prospective cohort study.

^b^Including antithrombosis stockings, intermittent pneumatic compression devices, and foot-pumps.

^c^Including aspirin, clopidogrel, dipyridamole, prasugrel, ticagrelor, ticlopidine, cilostazol, abciximab, eptifibatide, tirofiban, as well as thromboxane inhibitors, thromboxane synthase inhibitors, thromboxane receptor antagonists, and terutroban.

^d^Either single-surgeon series; single center, not single surgeon; multicenter in one country; international multicenter.

^e^Including warfarin, low molecular weight heparin, low dose unfractioned heparin, rivaroxaban, dabigatran, apixaban, fondaparinux, and idraparinux.

To assess applicability and representativeness of each study and to assess heterogeneity between estimates, we recorded the mean age of the study population and proportion of patients with malignant disease. When there were clear outliers, with atypical populations for either of these factors, we excluded those studies.

### Analysis

#### Choosing best estimate

We used the median value of estimates from studies with the lowest risk of bias to estimate baseline risk of VTE and bleeding requiring reoperation. The reason we chose the median as opposed to the pooled estimates across studies is that even the largest studies are likely to have factors idiosyncratic to that population and setting that will influence risks of both thrombosis and bleeding. There is little reason, given these idiosyncratic factors, that larger studies should have more weight than smaller studies. Under these circumstances, the median is likely to provide better estimates of typical risk than is the pooled estimate [18].

#### Modeling risk of VTE over time

**Figure 1:**
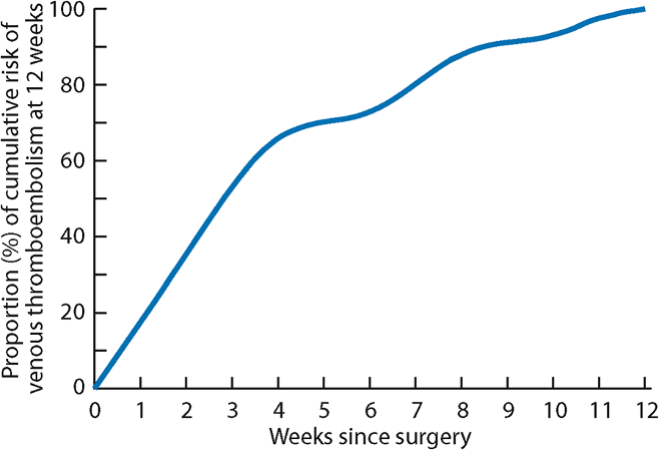
**Proportion of cumulative risk (%) of venous thromboembolism by week since surgery during the first 12 post-operative weeks** [19] **,** [20] **.**

**Figure 2:**
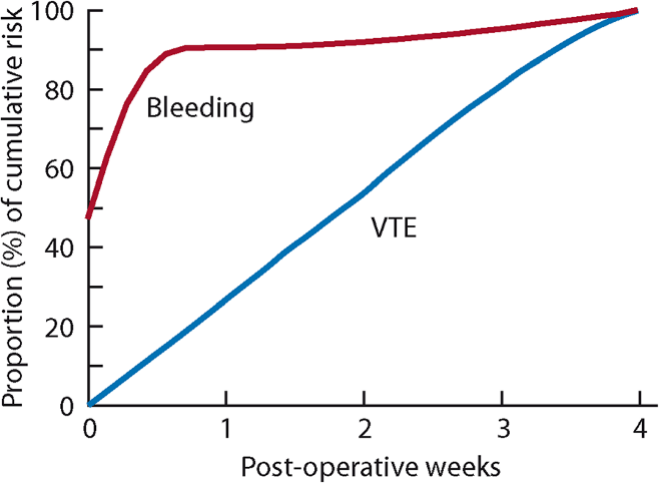
**Proportion of cumulative risk (%) of venous thromboembolism (VTE) and major bleeding by week since surgery during the first 4 post-operative weeks** [19] **,** [20] **,** [24] **.**

We provided procedure-stratified estimates for both risk of VTE and bleeding requiring reoperation in urological surgery. We chose one and four-week time frames for estimates of risks of thrombosis and bleeding because these are feasible and frequently chosen time frames for, respectively, shorter and longer term prophylaxis. For studies that did not report VTE estimates using these intervals, we modeled estimates—based on a literature search (Additional file 3)—using large-scale population-based studies (Amin, Beral, and Sweetland, personal communications) [19, 20] that have provided data regarding the timing of post-surgical VTE. These results are consistent with recent report using nationwide cystectomy data from the United States [21]. To calculate absolute risk of VTE by post-operative day, we calculated the mean values (of VTE risk) from the available studies [19, 20]. After assessing mean values for both studies and when calculating the final model for VTE (Figures 1 and 2), interpolated values were calculated using natural cubic spline interpolation [22] and R data analysis language [23]. When creating the model for timing of VTE (Figure 1), we used the thromboprophylaxis estimates from the population-based US study [17] where 81.4% had used either mechanical or pharmacological prophylaxis until discharge (i.e., 81.4% used for median of 4.5 days) and that 1.5% used from discharge (median discharge time 4.5 days) until median time of 35 days.

#### Modeling risk of bleeding over time

For studies that did not report their bleeding estimates at 4 weeks, we modeled timing of bleeding using data from the placebo arm of a large pragmatic RCT [24]. Ninety percent of the 30-day bleeding events happen during the first week after surgery (and indeed, approximately 75% in the first 2 days), so that only a small proportion of bleeding happens after the first post-operative week (Figure 2). For studies that provided bleeding data for a longer period than 30 days (but not more than 90 days), we therefore assumed a constant risk of bleeding beyond the first post-operative week, so for example, the day 30 rates were calculated at 80% of the rates at day 90 (Figure 2).

#### Calculating risks

We extracted information from contemporary observational studies. However, one of the challenges of these reviews relates to the decreasing incidence of VTE as surgical techniques have improved and early mobilization has become a standard of care [11–13]. Furthermore, in the recent studies representing current rates of VTE, patients often received prophylaxis. How then to estimate baseline risk?

To adjust estimates of baseline risk for use of prophylaxis, we used information from RCTs and meta-analyses of RCTs about the relative risk of VTE and bleeding among those who received prophylaxis [1, 24–26]. Specifically, we used estimates from a meta-analysis of RCTs in urology, general surgery, gynecology, and gastrointestinal surgery that concluded that anticoagulants (such as low molecular weight heparin, see Table 2) reduce the relative risk of VTE by 50% [1] and increases the relative risk of major bleeding by 50%. We used data from a meta-analysis of RCTs in orthopedic surgery [26] for our estimate that direct oral anticoagulants (Table 2) had similar effects on both VTE and bleeding as low molecular weight heparin. Based on a meta-analysis of RCTs in urology, general surgery, gynecology, and gastrointestinal surgery, we estimated the same efficacy of 50% VTE risk reduction for mechanical prophylaxis (Table 2) [1]. However, we had low certainty in the estimates for mechanical prophylaxis because studies used surrogate outcomes, had very few events, unblinded patients and assessors, and provided almost no information on intermittent pneumatic compression. Hence, we did not consider combination therapy as offering more protection than heparin alone when adjusting for prophylaxis in the baseline risk estimates of VTE. Finally, we assumed that aspirin (and other antiplatelet drugs, see Table 2) reduces the relative risk of VTE by 30% and increases the relative risk of major bleeding by 20%, based on two large pragmatic surgical trials [24, 25].

We then adjusted the reported risk by multiplying the relative risk by the reported risk in the fraction of patients who received prophylaxis. For instance, a study in which all patients received anticoagulant prophylaxis for 4 weeks or 28 days (time point of our primary outcome) showed a VTE risk of 1%, assuming that the relative risk reduction with prophylaxis is approximately 50%, we inferred that the included patients would have experienced a 2% risk of VTE had they not received prophylaxis.

Similarly, in the same study, the bleeding risk was 3%, because anticoagulant prophylaxis increases the risk of bleeding by approximately 50%, we inferred a bleeding risk of 2% without prophylaxis. Thromboprophylaxis was often used for less than 4 weeks. In those circumstances, we estimated its impact using same rationale but also considered the duration of thromboprophylaxis by using our models (Figure 2).

If the study did not provide estimates of VTE but only DVT and/or PE, we calculated the risk of VTE using the following approach. We reviewed data from studies that reported DVT, PE, and VTE totals and estimated the overlap (i.e., patients with both DVT and PE) from these studies. We then applied the degree of overlap to estimate VTE frequency in trials that provided only separate reports of DVT and/or PE.

#### Stratifying the risk of VTE according to patient risk factors

After assessing the baseline risk of VTE for each procedure, we stratified the risk by patient risk factors. We conducted a literature search addressing VTE risk factors in the context of urology, general surgery, gynecology, and gastrointestinal surgery (Additional file 4). We developed a very simple model for VTE risk based on the studies reporting the most relevant and compelling evidence [27–35]. Risk factors included 1) age more than 75 years, 2) obesity (body mass index of 35 or more), and 3) VTE in a first degree relative (parents, full siblings, or children)—all of these increase the risk approximately twofold. The most important risk factor was prior VTE, with risk ratio of approximately 4. We assumed that patients with any combination of two or more risk factors had a risk ratio of 4. Using these risk factors, we then categorized risk of VTE as low, medium (risk ratio of 2), and high risk (risk ratio of 4).

To calculate estimates of absolute risks for these groups, for each procedure, we estimated the proportion of patients having each of the risk factors using eligible studies. For both age and body mass index, we used the median value and median SD for estimating risk and assumed a normal distribution. We calculated the proportion of those with history of VTE based on large population-based study [35].

Our search did not reveal studies demonstrating convincing and replicable risk factors for bleeding (Additional file 5). Therefore, we did not stratify bleeding risk by patient specific factors.

#### Case fatality

We also estimated case fatality rates for VTE and bleeding requiring reoperation. For case fatality of VTE, we divided the number of fatal PE by the number of symptomatic VTE using studies that provided both estimates. Correspondingly, for case fatality of bleeding requiring reoperation, we divided the number of fatal bleeding by the number of bleeding requiring reoperation.

## Discussion

Our methods meet the criteria for rigorous systematic reviews. We specified explicit eligibility criteria, conducted comprehensive searches (not only for baseline risk but also for timing of complications and patient risk factors), and assessed risk of bias using criteria specific to this review. In addition, teams of two reviewers independently assessed eligibility and risk of bias and extracted data, a third reviewer adjudicated discrepancies, and we sent our consensus of data extraction to the original authors of each article for confirmation or correction. We considered, for the first time in urology, risk of VTE and bleeding separately for each procedure including surgical technique (for instance, open vs. robotic and radical vs. partial). We took into account length of follow-up, use of thromboprophylaxis, and patient risk factors.

Our reviews have limitations. Our analyses reflect published data and we cannot completely exclude publication bias. Because reporting of surgical complications is sporadic and *ad hoc*, it is possible that literature is biased towards underreporting of events. This is especially true regarding single-surgeon series: individual surgeons experiencing high rates of complications are unlikely to publish their results. Secondly, due to poor reporting standards (both indexing and abstract information), our search likely missed some relevant studies.

We have carefully addressed the many challenges involved in generating best estimates of risk of VTE and bleeding in the absence of antithrombotic prophylaxis in patients undergoing urological surgery. Clinicians can therefore look to the systematic reviews in this series as providing the best current estimates of the risk of both symptomatic VTE and bleeding requiring reoperation in urological procedures. Guideline panels will find these estimates helpful when formulating recommendations for VTE prophylaxis in urological surgery. Moreover, these reviews provide guidance for best practices in systematic reviews of observational data—particularly with regard to surgical complications.

## Electronic supplementary material

Additional file 1: **Search history for baseline risk of VTE.** (DOCX 32 KB)

Additional file 2: **Search history for baseline risk of major bleeding/bleeding requiring reoperation.** (DOCX 32 KB)

Additional file 3: **Search history for modeling of risk for venous thromboembolism after surgery.** (DOCX 29 KB)

Additional file 4: **Search history for patient related risk factors of venous thromboembolism after surgery.** (DOCX 29 KB)

Additional file 5: **Search history for patient related risk factors of major bleeding/bleeding requiring reoperation after surgery.** (DOCX 29 KB)

## Abbreviations

CLUE: clinical urology and epidemiology
DVT: deep vein thrombosis
EAU: European Association of Urology
GRADE: grading of recommendations assessment, development and evaluation
PICO: patient, intervention, comparator and outcome
PE: pulmonary embolism
RCT: randomized controlled trial
ROTBUS: risk of thrombosis and bleeding in urological surgery
VTE: venous thromboembolism.
