# Supplementary material for: Systematic reviews of observational studies of risk of thrombosis and bleeding in urological surgery (ROTBUS): introduction and methodology
Source: Syst Rev. 2014 Dec 23;3:150. doi: 10.1186/2046-4053-3-150 (PMC4307154; doi:10.1186/2046-4053-3-150)
Supplement: Supplementary file 1 — Additional file 1: Search history for baseline risk of VTE. (DOCX 32 KB) [file 13643_2014_318_MOESM1_ESM.docx]

**Additional file 1.** Search history for baseline risk of VTE.

Database: Ovid MEDLINE(R) In-Process & Other Non-Indexed Citations and Ovid MEDLINE(R) <1946 to April 10, 2014>

Search Strategy:

--------------------------------------------------------------------------------

1 exp Embolism/ or embolism.mp.

2 exp Thromboembolism/ or Thromboembolism.mp.

3 Venous Thromboembolism.mp. or exp Venous Thromboembolism/

4 Thrombophlebitis.mp. or exp Thrombophlebitis/

5 thromboprophylaxis.mp.

6 1 or 2 or 3 or 4 or 5

7 Urologic Surgical Procedures.mp. or exp Urologic Surgical Procedures/

8 Cystectomy.mp.

9 Cystoscopy.mp.

10 Cystostomy.mp.

11 Cystotomy.mp.

12 Kidney Transplantation.mp.

13 Nephrectomy.mp.

14 Ureteroscopy.mp.

15 Urinary Diversion.mp.

16 Nephrostomy, Percutaneous.mp.

17 Circumcision, Male.mp.

18 Orchiectomy.mp.

19 Orchiopexy.mp.

20 Penile Implantation.mp.

21 Prostatectomy.mp.

22 Vasectomy.mp.

23 Vasovasostomy.mp.

24 7 or 8 or 9 or 10 or 11 or 12 or 13 or 14 or 15 or 16 or 17 or 18 or 19 or 20 or 21 or 22 or 23

25 Adrenalectomy.mp. or exp Adrenalectomy/

26 Nephroureterectomy.mp.

27 Renal arterial grafts.mp.

28 Splenorenal arterial bypass.mp.

29 Hepatorenal arterial bypass.mp.

30 Adrenalectomy.mp. or exp Adrenalectomy/

31 Pyeloplasty.mp.

32 Pyeloureteroplasty.mp.

33 Symphysiotomy of horseshoe kidney.mp.

34 Nephrostomy.mp.

35 Pyelostomy.mp.

36 Nephrostomy, Percutaneous.mp. or exp Nephrostomy, Percutaneous/

37 Endopyelotomy.mp.

38 Pyelonephrolithotomy.mp.

39 Ureterocalicostomy.mp.

40 Ureterostomy.mp. or exp Ureterostomy/

41 Ureterolithotomy.mp.

42 Psoas hitch.mp.

43 Boari flap.mp.

44 Ureterocolic anastomosis.mp.

45 Urinary Diversion.mp. or exp Urinary Diversion/

46 Ureteroileal conduit.mp.

47 Ureteroureterostomy.mp.

48 Transureteroureterostomy.mp.

49 Ureterovaginal fistula repair.mp.

50 Repair of ureteral injuries.mp.

51 Ureterectomy.mp.

52 Ureteral reimplantation.mp.

53 Megaureter repair.mp.

54 Ureteroscopy.mp. or exp Ureteroscopy/

55 Laparoscopic surgery of the ureter.mp.

56 Retroperitoneal lymph node dissection.mp.

57 Ureterolysis for retroperitoneal fibrosis.mp.

58 Intracorporeal lithotripsy.mp.

59 Lithotripsy/ or Lithotripsy.mp.

60 Lithotripsy, Laser.mp. or exp Lithotripsy, Laser/

61 ESWL.mp.

62 Extracorporeal shock wave lithotripsy.mp.

63 Excision of urachus.mp.

64 Repair of ruptured bladder.mp.

65 Vesical diverticulectomy.mp.

66 Cystolithotomy.mp.

67 Vesical diverticulectomy.mp.

68 Cystolithotomy.mp.

69 Closure of fistula.mp.

70 Transurethral resection of bladder tumor.mp.

71 TURBT.mp.

72 Laser treatment of bladder cancer.mp.

73 Augmentation cystoplasty.mp.

74 Sacral nerve stimulation.mp.

75 Vesicolithotomy.mp.

76 Meatotomy.mp.

77 Excision of stricture.mp.

78 Diverticulectomy.mp.

79 Artificial urinary sphincter implant.mp.

80 Urethroplasty.mp.

81 Urethrectomy.mp.

82 Repair of urethral fistula.mp.

83 Fistula repair.mp.

84 Dilation of stricture.mp.

85 Endoscopic treatment of urethral strictures.mp.

86 Trans urethral resection of the prostate.mp. or exp "Transurethral Resection of Prostate"/

87 TURP.mp.

88 Needle biopsy of the prostate.mp.

89 Prostatic stenting.mp.

90 Orchiopexy.mp. or exp Orchiopexy/

91 Herniorrhaphy.mp. or exp Herniorrhaphy/

92 Hydrocelectomy.mp.

93 Varicocelectomy.mp.

94 Spermatocelectomy.mp.

95 Microscopic vasovasostomy.mp.

96 Microscopic vasoepididymostomy.mp.

97 Management of male factor infertility.mp.

98 Management of testicular torsion.mp.

99 Colporrhaphy.mp.

100 Pubovaginal slings.mp.

101 Sacrocolpopexy.mp.

102 Sacrohysteropexy.mp.

103 Sacrospinous fixation.mp.

104 25 or 26 or 27 or 28 or 29 or 30 or 31 or 32 or 33 or 34 or 35 or 36 or 37 or 38 or 39 or 40 or 41 or 42 or 43 or 44 or 45 or 46 or 47 or 48 or 49 or 50 or 51 or 52 or 53 or 54 or 55 or 56 or 57 or 58 or 59 or 60 or 61 or 62 or 63 or 64 or 65 or 66 or 67 or 68 or 69 or 70 or 71 or 72 or 73 or 74 or 75 or 76 or 77 or 78 or 79 or 80 or 81 or 82 or 83 or 84 or 85 or 86 or 87 or 88 or 89 or 90 or 91 or 92 or 93 or 94 or 95 or 96 or 97 or 98 or 99 or 100 or 101 or 102 or 103

105 24 or 104

106 6 and 105

107 limit 106 to yr="2000 -Current"
