# Supplementary material for: Systematic reviews of observational studies of risk of thrombosis and bleeding in urological surgery (ROTBUS): introduction and methodology
Source: Syst Rev. 2014 Dec 23;3:150. doi: 10.1186/2046-4053-3-150 (PMC4307154; doi:10.1186/2046-4053-3-150)
Supplement: Supplementary file 2 — Additional file 2: Search history for baseline risk of major bleeding/bleeding requiring reoperation. (DOCX 32 KB) [file 13643_2014_318_MOESM2_ESM.docx]

**Additional file 2.** Search history for baseline risk of major bleeding/bleeding requiring reoperation.

Database: Ovid MEDLINE(R) In-Process & Other Non-Indexed Citations and Ovid MEDLINE(R) <1946 to April 10, 2014>

Search Strategy:

--------------------------------------------------------------------------------

1 Urologic Surgical Procedures.mp. or exp Urologic Surgical Procedures/

2 Cystectomy.mp.

3 Cystoscopy.mp.

4 Cystostomy.mp.

5 Cystotomy.mp.

6 Kidney Transplantation.mp.

7 Nephrectomy.mp.

8 Ureteroscopy.mp.

9 Urinary Diversion.mp.

10 Nephrostomy, Percutaneous.mp.

11 Circumcision, Male.mp.

12 Orchiectomy.mp.

13 Orchiopexy.mp.

14 Penile Implantation.mp.

15 Prostatectomy.mp.

16 Vasectomy.mp.

17 Vasovasostomy.mp.

18 1 or 2 or 3 or 4 or 5 or 6 or 7 or 8 or 9 or 10 or 11 or 12 or 13 or 14 or 15 or 16 or 17

19 Nephroureterectomy.mp.

20 Renal arterial grafts.mp.

21 Splenorenal arterial bypass.mp.

22 Hepatorenal arterial bypass.mp.

23 Pyeloplasty.mp.

24 Pyeloureteroplasty.mp.

25 Symphysiotomy of horseshoe kidney.mp.

26 Nephrostomy.mp.

27 Pyelostomy.mp.

28 Nephrostomy, Percutaneous.mp. or exp Nephrostomy, Percutaneous/

29 Endopyelotomy.mp.

30 Pyelonephrolithotomy.mp.

31 Ureterocalicostomy.mp.

32 Ureterostomy.mp. or exp Ureterostomy/

33 Ureterolithotomy.mp.

34 Psoas hitch.mp.

35 Boari flap.mp.

36 Ureterocolic anastomosis.mp.

37 Urinary Diversion.mp. or exp Urinary Diversion/

38 Ureteroileal conduit.mp.

39 Ureteroureterostomy.mp.

40 Transureteroureterostomy.mp.

41 Ureterovaginal fistula repair.mp.

42 Repair of ureteral injuries.mp.

43 Ureterectomy.mp.

44 Ureteral reimplantation.mp.

45 Megaureter repair.mp.

46 Ureteroscopy.mp. or exp Ureteroscopy/

47 Laparoscopic surgery of the ureter.mp.

48 Retroperitoneal lymph node dissection.mp.

49 Ureterolysis for retroperitoneal fibrosis.mp.

50 Intracorporeal lithotripsy.mp.

51 Lithotripsy/ or Lithotripsy.mp.

52 Lithotripsy, Laser.mp. or exp Lithotripsy, Laser/

53 ESWL.mp.

54 Extracorporeal shock wave lithotripsy.mp.

55 Excision of urachus.mp.

56 Repair of ruptured bladder.mp.

57 Vesical diverticulectomy.mp.

58 Cystolithotomy.mp.

59 Vesical diverticulectomy.mp.

60 Cystolithotomy.mp.

61 Closure of fistula.mp.

62 Transurethral resection of bladder tumor.mp.

63 TURBT.mp.

64 Laser treatment of bladder cancer.mp.

65 Augmentation cystoplasty.mp.

66 Sacral nerve stimulation.mp.

67 Vesicolithotomy.mp.

68 Meatotomy.mp.

69 Excision of stricture.mp.

70 Diverticulectomy.mp.

71 Artificial urinary sphincter implant.mp.

72 Urethroplasty.mp.

73 Urethrectomy.mp.

74 Repair of urethral fistula.mp.

75 Fistula repair.mp.

76 Dilation of stricture.mp.

77 Endoscopic treatment of urethral strictures.mp.

78 Trans urethral resection of the prostate.mp. or exp "Transurethral Resection of Prostate"/

79 TURP.mp.

80 Needle biopsy of the prostate.mp.

81 Prostatic stenting.mp.

82 Orchiopexy.mp. or exp Orchiopexy/

83 Hydrocelectomy.mp.

84 Varicocelectomy.mp.

85 Spermatocelectomy.mp.

86 Microscopic vasovasostomy.mp.

87 Microscopic vasoepididymostomy.mp.

88 Management of male factor infertility.mp.

89 Management of testicular torsion.mp.

90 Colporrhaphy.mp.

91 Pubovaginal slings.mp.

92 Sacrocolpopexy.mp.

93 Sacrohysteropexy.mp.

94 Sacrospinous fixation.mp.

95 19 or 20 or 21 or 22 or 23 or 24 or 25 or 26 or 27 or 28 or 29 or 30 or 31 or 32 or 33 or 34 or 35 or 36 or 37 or 38 or 39 or 40 or 41 or 42 or 43 or 44 or 45 or 46 or 47 or 48 or 49 or 50 or 51 or 52 or 53 or 54 or 55 or 56 or 57 or 58 or 59 or 60 or 61 or 62 or 63 or 64 or 65 or 66 or 67 or 68 or 69 or 70 or 71 or 72 or 73 or 74 or 75 or 76 or 77 or 78 or 79 or 80 or 81 or 82 or 83 or 84 or 85 or 86 or 87 or 88 or 89 or 90 or 91 or 92 or 93 or 94

96 18 or 95

97 hemorrhage/ or blood loss, surgical/ or exsanguination/ or hematocele/ or hematoma/ or hemoperitoneum/ or postoperative hemorrhage/ or shock, hemorrhagic/

98 96 and 97

99 limit 98 to "prognosis (maximizes sensitivity)"

100 limit 99 to yr="2000 -Current"
