# Supplementary material for: Systematic reviews of observational studies of risk of thrombosis and bleeding in urological surgery (ROTBUS): introduction and methodology
Source: Syst Rev. 2014 Dec 23;3:150. doi: 10.1186/2046-4053-3-150 (PMC4307154; doi:10.1186/2046-4053-3-150)
Supplement: Supplementary file 3 — Additional file 3: Search history for modeling of risk for venous thromboembolism after surgery. (DOCX 29 KB) [file 13643_2014_318_MOESM3_ESM.docx]

**Additional file 3.** Search history for modeling of risk for venous thromboembolism after surgery.

Database: Ovid MEDLINE(R) In-Process & Other Non-Indexed Citations and Ovid MEDLINE(R) <1946 to August 30, 2014>

Search Strategy:

--------------------------------------------------------------------------------

1 exp Embolism/

2 exp Thromboembolism/

3 exp Venous Thrombosis/

4 exp Thrombophlebitis/

5 1 or 2 or 3 or 4

6 exp Colorectal Surgery/

7 exp Gynecology/

8 exp Urology/

9 exp General Surgery/

10 inpatient surgery.mp.

11 6 or 7 or 8 or 9 or 10

12 prognosis.mp. or exp Prognosis/

13 time factors.mp. or exp Time Factors/

14 Forecasting.mp. or exp Forecasting/

15 outcome prediction.mp.

16 exp Diagnosis/ or diagnosis.mp.

17 12 or 13 or 14 or 15 or 16

18 5 and 11 and 17

19 limit 18 to yr="2000 -Current"
