# Supplementary material for: Systematic reviews of observational studies of risk of thrombosis and bleeding in urological surgery (ROTBUS): introduction and methodology
Source: Syst Rev. 2014 Dec 23;3:150. doi: 10.1186/2046-4053-3-150 (PMC4307154; doi:10.1186/2046-4053-3-150)
Supplement: Supplementary file 4 — Additional file 4: Search history for patient related risk factors of venous thromboembolism after surgery. (DOCX 29 KB) [file 13643_2014_318_MOESM4_ESM.docx]

**Additional file 4.** Search history for patient related risk factors of venous thromboembolism after surgery.

Database: Ovid MEDLINE(R) In-Process & Other Non-Indexed Citations and Ovid MEDLINE(R) <1946 to August 30, 2014>

Search Strategy:

--------------------------------------------------------------------------------

1 exp Embolism

2 exp Thromboembolism

3 exp Venous Thrombosis

4 exp Thrombophlebitis

5 1 or 2 or 3 or 4

6 exp Colorectal Surgery

7 exp General Surgery

8 exp Gynecology

9 exp Urology

10 6 or 7 or 8 or 9

11 5 and 10

12 limit 11 to yr="2000 -Current"
