# Supplementary material for: Systematic reviews of observational studies of risk of thrombosis and bleeding in urological surgery (ROTBUS): introduction and methodology
Source: Syst Rev. 2014 Dec 23;3:150. doi: 10.1186/2046-4053-3-150 (PMC4307154; doi:10.1186/2046-4053-3-150)
Supplement: Supplementary file 5 — Additional file 5: Search history for patient related risk factors of major bleeding/bleeding requiring reoperation after surgery. (DOCX 29 KB) [file 13643_2014_318_MOESM5_ESM.docx]

**Additional file 5.** Search history for patient related risk factors of major bleeding/bleeding requiring reoperation after surgery.

Database: Ovid MEDLINE(R) In-Process & Other Non-Indexed Citations and Ovid MEDLINE(R) <1946 to August 30, 2014>

Search Strategy:

--------------------------------------------------------------------------------

1 hemorrhage/ or blood loss, surgical/ or exsanguination/ or hematocele/ or hematoma/ or hemoperitoneum/ or postoperative hemorrhage/ or shock, hemorrhagic/

2 exp Colorectal Surgery

3 exp General Surgery

4 exp Gynecology

5 exp Urology

6 2 or 3 or 4 or 5

7 1 and 6

8 limit 7 to yr="2000 -Current"
